# Fabrication of polyvinyl alcohol/soy protein isolate-based composite nanofilm for preserving Chinese cabbage

**DOI:** 10.1016/j.fochx.2025.103048

**Published:** 2025-09-17

**Authors:** Lanlan Wei, Yanyan Yang, Ziyi Qin, Fuqiang Liang, Hong Xie

**Affiliations:** a School of Public Health, Department of Biochemistry and Molecular Biology, School of Laboratory Medicine, Anhui Province Key Laboratory of Immunology in Chronic Diseases, Bengbu Medical College, Bengbu 233030, China; b College of Food Science and Engineering, Collaborative Innovation Center for Modern Grain Circulation and Safety, Nanjing University of Finance and Economics, Nanjing 210023, China; c College of Food Engineering, Anhui Science and Technology University, Chuzhou, 233100, China

**Keywords:** Electrospinning, Ε-Polylysine, Polyvinyl alcohol, Soy protein isolate, Active packaging materials

## Abstract

A composite film based on a polyvinyl alcohol (PVA)/soy protein isolate (SPI) loaded with ε-polylysine (ε-PL) was prepared via electrospinning and utilized to preserve the freshness of Chinese cabbage. The active films were characterized using Fourier transform infrared spectroscopy, X-ray diffraction, scanning electron microscopy, and differential scanning calorimeter. Further molecular dynamic simulations results confirmed that PVA can spontaneously form stable complexes with SPI, which was mainly driven by the combined contribution of van der Waals interactions, electrostatic interactions and non-polar solvation energy. The binding of PVA via its hydroxyl groups interactions with many residues of protein surface resulted in the change of protein conformation and solvent assessable surface area. The SPI/PVA nanofiber film loaded with 8 % ε-PL exhibited stronger antibacterial activity against *Escherichia coli* (*E. coli*) and *Staphylococcus aureus* (*S. aureus*), with inhibition zone diameters measuring 13.93 ± 0.14 mm and 17.54 ± 0.17 mm, respectively. Its density was measured at 0.32 ± 0.03 g/cm^3^, with a swelling ratio of 199.36 ± 23.54 %, solubility of 25.87 ± 0.31 %, and a water vapor permeability of 0.53 ± 0.07 g·mm/m^2^·h·pa. The utilization of 8 % ε-PL-loaded SPI/PVA nanofiber film effectively prevented the weight losses, chlorophyll, carotenoids, and lutein reduction of Chinese cabbages. Overall, this study uncovered the molecular mechanisms underlying the successful fabrication of ε-PL/SPI/PVA nanofiber film and further demonstrated its potential for Chinese cabbages preservation.

## Highlights

• The PVA/SPI composite nanofiber film embedded with ε-PL were prepared.
• The PVA/SPI/ε-PL film have potential in the preservation of Chinese cabbage.
• The films exhibited good antibacterial activity against *E. coli* and *S. aureus*.
• Further molecular dynamic simulation confirmed that PVA can spontaneously form stable complexes with SPI.

## 1 Introduction

Chinese cabbage (*Brassica rapa* L. *ssp. pekinensis*), one of the most economically important and abundant green leafy vegetables, is extensively cultivated and consumed worldwide due to its high yield, nutritional value, special texture, and extended supply period (Zhang et al., 2021). However, it is highly susceptible to water loss and quality deterioration during postharvest storage, unlike many other vegetables, due to its high moisture content and heightened metabolic activity (Liu et al., 2021). As a result, producers face severe challenges in maintaining the freshness and quality of Chinese cabbage, which is crucial for ensuring a smooth flow through the supply chain. The utilization of polymeric film packaging can effectively control environmental conditions during storage, thereby reducing the rate of physiological activities such as respiration, transpiration, and other metabolic processes (Yun et al., 2024; Tsai et al., 2025). Traditional petrochemical-based plastics have long been used for food packaging materials, but they contribute significantly to environmental pollution. In recent years, biopolymers derived from proteins, lipids, and polysaccharides have been increasingly used to develop eco-friendly food packaging materials. In addition to their biocompatibility, biodegradability, renewability, abundance, and affordability (Gupta et al., 2022; Jayakody et al., 2022), these biopolymer-based films can integrate antioxidants and antibacterial agents. These advantages facilitate the production of active food packaging without introducing hazardous substances into food (Moeini et al., 2021).

Soy protein isolate (SPI), a byproduct of soybean oil production, has received an increase interest as a biopolymer film material in food industry. In addition to its high protein content (>90 %), SPI has many advantages, including high abundance, nontoxicity, biocompatibility, and critically, excellent film-forming properties (Chen et al., 2025; Kang et al., 2023). Consequently, SPI-based active packaging materials have attracted considerable attention owing to their antioxidant and antimicrobial activities (Koshy et al., 2022; Tao et al., 2022; Hu et al., 2023;). However, compared with traditional plastic packaging, SPI-based films exhibit limitation in key physicochemical properties, particularly barrier properties, mechanical strength, and thermal stability, which restrict their broader application in food packaging (Chen et al., 2025). Creative to develop new strategies to enhance the hydrophilicity of SPI. Polyvinyl alcohol (PVA) is a biodegradable synthetic polymer that is extensively used as a food packaging material owing to its unique characteristics, including biodegradability, chemical resistance, low oxygen permeability, and film-forming ability (Ma et al., 2024). Electrospinning is a versatile technology that enables the easy and flexible fabrication of nanofiber films (Jovanska et al., 2021). The nanofiber films produced through electrospinning are characterized by large surface area/volume ratio, high porosity and loading capacity (Pan et al., 2022; Tavassoli et al., 2025). Consequently, these excellent properties enable electrospun nanofiber films suitable for the sustained delivery and release of natural antibacterial agents, thereby expanding their application in active food packaging.

ε-polylysine (ε-PL), a cationic natural and broad-spectrum antimicrobial peptide composed of 25–30 l-lysine residues, is well-known for its antibacterial activity, safety and environmentally friendly (Hu et al., 2023). It has several unique properties, including high water solubility, nontoxicity, and biodegradability (Liao et al., 2023). Nevertheless, producing pure ε-PL films is challenging due to its poor flexibility and stability. In recent years, researchers have explored the incorporation of ε-PL into diverse edible films to overcome these limitations (Cheng et al., 2022). Therefore, in this study, SPI and PVA were blended via electrospinning to develop an active nanofilm loaded with ε-polylysine (ε-PL). Furthermore, the physical properties and formation mechanisms of the nanofilm were investigated, along with its efficacy in preserving the freshness of Chinese cabbage.

## 2 Materials and methods

### 2.1 Materials and reagents

PVA was purchased from Shanghai Aladdin Biochemical Technology Co., Ltd., and potassium bromide (KBr) was purchased from Sinopharm chemical reagent Co., Ltd. ε-polylysine and SPI were purchased from Shanghai Yuan-Ye Bio-Technology Co., Ltd. Both nutritional agar and plate counting agar were collected from Shanghai BW Technology Co., Ltd. *Escherichia coli (ATCC·25922)* and *Staphylococcus aureus (ATCC·6538)* were acquired from Shanghai Gaoxin Chemical Glass Co., Ltd. The Chinese cabbages in this study were purchased from the local supermarket named Shanghai Green (Jingguan NO.1) and the whole Chinese cabbages were fully mature with almost the same weight and no mechanical damage.

### 2.2 Preparation of nanofiber film

PVA (10 g) was added to 100 mL of distilled water and stirred at 90 °C in a water bath for 2 h at 5000 rpm. Meanwhile, SPI (3.0 g) was dissolved in 100 mL of distilled water and stirred at 80 °C water bath for 20 min at 5000 rpm. The pH of the SPI solution was adjusted to 9 using 2 % NaOH. Once the two solutions cooled to 50 °C, they were mixed, and the final concentration of ε-PL in the SPI/PVA mixed system was set to 0, 2, 4, 6, and 8 % (*w*/*v*). Then, 5.0 mL of electrospinning solution was loaded into a syringe filled with a No. 5 needle. The flow rate of the electrospinning solution was set at 0.8 mL h^−1^, and the voltage and the distance between the collector plate and syringe were 16 kV and 14–18 cm, respectively. The experiment was conducted in an environment at 25 °C with approximately 50 % humidity.

### 2.3 Characterization of nanofiber film

#### 2.3.1 Scanning electron microscopy (SEM)

The nanofiber film was pasted on a glass slide with conductive adhesive, and the surface of nanofiber film surface was characterized using SEM (Supra55, Zeiss, Germany) at a voltage of 10 kV. Prior to testing, the film surface was sprayed with gold under vacuum.

#### 2.3.2 Fourier transform infrared spectroscopy (FTIR)

The absorption spectra of the nanofiber films were recorded using an FTIR spectrometer (Nicolet S I10 Spectrometer, Thermo Fisher, USA) over a range of 4000–400 cm^−1^ with a resolution of 4 cm^−1^.

#### 2.3.3 X-ray diffraction (XRD)

The XRD analysis of films was measured using an XRD system (Persee General, China) installed with the Cu Kα radiation, and the angle range (2θ) was set from 5 to 50° at a scan rate of 5°min^−1^.

#### 2.3.4 Molecular dynamic simulation

The three-dimensional structures of glycinin (PDB ID: 1FXZ) and *β*-conglycinin (1UIJ) were obtained from RCSB PDB database (https://www.rcsb.org/). The structure of PVA was generated according to a previous study with some modifications (Abdollahi et al., 2024). All-atom molecular dynamic simulations were performed using the Gromacs 2019.6 software package within Amber99sb-ildn force field. The initial simulation systems were constructed using Packmol by randomly placing a PVA and either a glycinin or β-conglycinin in a cubic box, with a minimum distance of 1.0 nm from the edge of the box. Then all the boxes were subsequently solvated using the TIP3P water model, followed by the addition of counterions (Na^+^ and Cl^−^) to neutral the simulation systems. Energy minimization, 100 ps NVT (number of particles, volume and temperature) and NPT (number of particles, pressure and temperature) equilibrations were performed subsequently, followed by 50 ns no-restrained production simulations. Then the results were analyzed and visualized by the combination of Gromacs and VMD. The gmx_MMPBSA program was used to calculate the MM/GBSA (Molecular Mechanics Generalized Born Surface Area) binding free energy (Valdés-Tresanco et al., 2021).

#### 2.3.5 Water vapor permeability (WVP)

To determine the WVP of the nanofiber films with a certain thickness (d), the films were cut into 8 × 8 cm (s) and sealed with paraffin in a weighing bottle containing two-thirds of anhydrous CaCl_2_. The weighing bottle was then placed in a desiccator with pure water. Finally, under a temperature of 25 °C and a pressure difference (ΔP) of 3168 Pa, the weight change (Δm) of the entire nanofiber film and the weighing bottle was measured every 2 h (t, s) until a constant weight was achieved. The WVP of the films was calculated as follows:

(1) $$\mathrm{WVP}=\frac{△m\times d}{A\times △t\times △p}$$

where the Δm is the weight change of the entire nanofiber film, d is the thickness, Δt is the time interval, Δp is vapor pressure difference, and A is the test area.

#### 2.3.6 Density, water content, solubility, and swelling of the composite nanofiber films

The initial weight of the film sample (m_0_) was recorded, and the sample was then dried at 105 °C until constant weight (m_1_) was achieved. After the sample was dried, the film sample was placed in a culture dish containing 30 mL of distilled water and stored at 25 °C for 24 h. Then, the moisture on the surface of the sample was gently wiped off with filter paper, and the sample was weighed (m_2_). Finally, the film sample was dried again at 105 °C until constant weight (m_3_) was achieved. The density, water content, solubility, and swelling were calculated as follows:

(2) $$\text{Density}=\frac{m_{0}}{s*d}$$

(3) $$\text{Solubility}=\frac{m_{1}-m_{3}}{m_{1}}$$

(4) $$\text{Swelling}=\frac{m_{2}-m_{1}}{m_{1}}$$

(5) $$\text{Water content}=\frac{m_{0}-m_{1}}{m_{0}}$$

### 2.4 Preservative effect of nanofiber film on Chinese cabbages

First, gram-positive bacteria, such as *Staphylococcus aureus,* and gram-negative bacteria, such as *Escherichia coli,* were selected and cultured in nutrient broth at 37 °C for 12 h under shaking conditions. Then, 100 μL of the bacterial solution was evenly spread on a solid medium. Then, a circular nanofiber film with a diameter of 6 mm was placed at the center of the culture medium and cultivated at 37 °C for 24 h. The inhibition zone around the nanofiber film was recorded using a digital micrometer, and the antibacterial test was repeated three times.

Chinese cabbages of similar weight and free from physical damage were selected for this research. They were thoroughly rinsed with distilled water to remove impurities and dried using filter paper. Then, a total of 60 g of Chinese cabbage leaves was packaged with PVA/SPI, 2 % ε-PL/PVA/SPI, 4 % ε-PL/PVA/SPI, 6 % ε-PL/PVA/SPI, and 8 % ε-PL/PVA/SPI nanofiber films. An unwrapped group was used as the control group. All the experimental groups, including both the packaged and control groups, were immediately stored at 4 ± 2 °C for 12 d, and different indices were measured at 0, 3, 6, 9, and 12 d.

#### 2.4.1 Color measurement

The color changes on the surface of Chinese cabbage leaves during 12 d storage period were evaluated. First, the main veins were avoided, and three points on the surface of the leaves were randomly selected from each group. Then, the L*, a*, and b* values of the leaves were determined using a colorimeter (NR110, 3nh Global, Shenzhen, China), ensuring that the same testing area of Chinese cabbage leaves was measured for each group.

#### 2.4.2 Weight loss

Weight loss in both the control and packaged Chinese cabbage groups were measured as follows: the initial weight (m_0_) and the final weight (m_1_) of the Chines cabbage at the end of the storage period were recorded using an analytical balance. The weight loss was calculated using the following equation:

(6) $$\text{Weight loss}=\frac{m_{0}-m_{1}}{m_{0}}\times 100\%$$

#### 2.4.3 Determination of chlorophyll, carotenoids, and lutein

A total of 0.2 g of Chinese cabbage leaf was extracted with 10 mL of 95 % ethanol under dark conditions for 8 h. After filtration, the supernatant was collected, and the absorbance values of the supernatant were measured at 470, 474, 485, 642, and 665 nm. Meanwhile, 95 % ethanol was used as the blank control. The concentrations of the total chlorophyll, carotenoids, and lutein can be calculated as follows:

(7) $$\text{Chlorophyll}\;a\;\left(\mathrm{mg}\;L^{-1}\right)=9.99\;A_{665}–0.087\;A_{642}$$

(8) $$\text{Chlorophyll}\;b\;\left(\mathrm{mg}\;L^{-1}\right)=17.7\;A_{642}–3.04\;A_{665}$$

(9) $$\text{Total chlorophyll content}\;\left(\mathrm{mg}\;L^{-1}\right)=\text{Chlorophyll}\;a+\text{Chlorophyll}\;b$$

(10) $$\text{Carotenoids}\;\left(\mathrm{mg}\;L^{-1}\right)=4.92\;A_{474}–0.0255\;\text{Chlorophyll}\;a\;\left(\mathrm{mg}\;L^{-1}\right)-0.225\;\text{Chlorophyll}\;b\;\left(\mathrm{mg}\;L^{-1}\right)$$

(11) $$\text{Lutein}\;\left(\mathrm{mg}\;L^{-1}\right)=10.2\;A_{470}–11.5\;A_{485}–0.0036\;\text{Chlorophyll}\;a\;\left(\mathrm{mg}\;L^{-1}\right)-0.652\;\text{Chlorophyll}\;b\;\left(\mathrm{mg}\;L^{-1}\right)$$

### 2.5 Statistical analysis

The data were analyzed using the analysis of variance to evaluate statistical significance between the samples of each group, expressed as the mean ± standard deviation. A probability value of *p* < 0.05 was regarded as statistically significant.

## 3 Results and discussion

### 3.1 Microstructure of composite nanofiber films

**Fig. 1:**
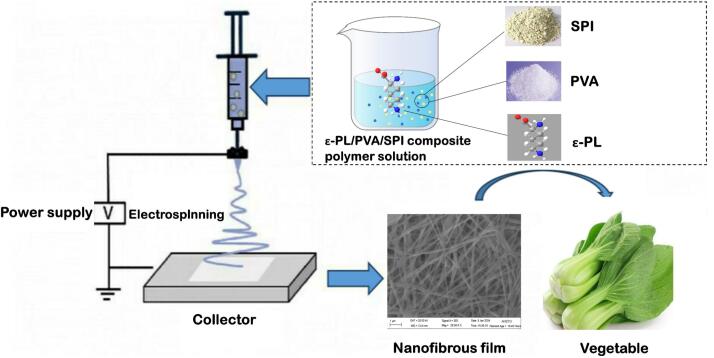
Synthesis of the nanofiber film and its application for preserving Chinese cabbage.

**Fig. 2:**
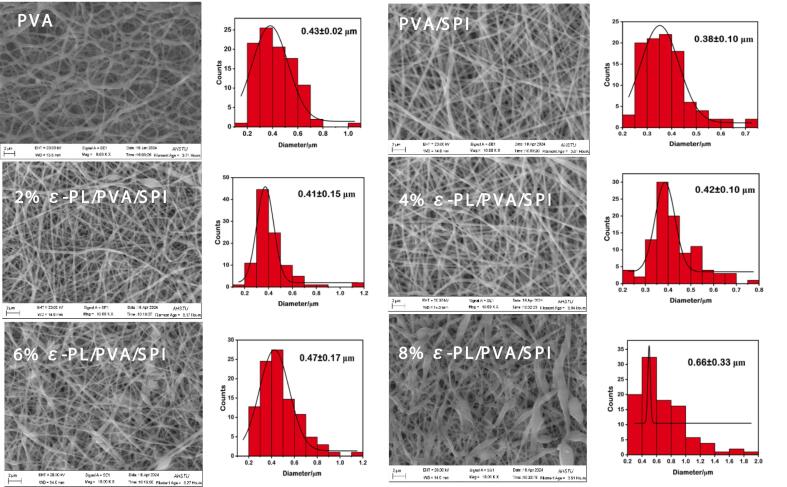
The SEM image of the different kind of electrospinning nanofiber films included PVA, PVA/SPI, 2 % ε-PL/PVA/SPI, 4 % ε-PL/PVA/SPI, 6 % ε-PL/PVA/SPI AND 8 % ε-PL/PVA/SPI.

The morphology of the fully prepared electrospinning nanofiber films is shown in Fig. 2, showing a disordered yet uniform diameter distribution, smooth surface, and consistent network structure with good continuity. The addition of ε-PL significantly altered the morphology and diameter of the nanofiber films. Previous studies have reported similar phenomena, which might be attributed to evaluated ε-PL concentrations increasing spinning solution viscosity (Lan et al., 2022; Zhao et al., 2025). This impedes jet fission at the needle tip and fiber stretching, resulting in an increase in the fiber diameter (Zhao et al., 2025). Despite these changes, the SEM results suggested that the film-forming ability remained largely unchanged. The results indicated that *ε*-PL was successfully incorporated into PVA/SPI nanofibers. (See Fig. 1.)

**Fig. 3:**
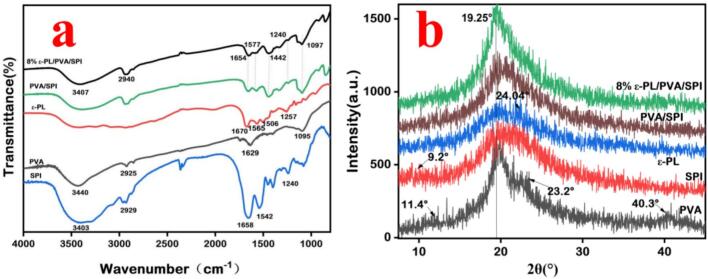
The FTIR (a) and XRD (b) patterns of the SPI, PVA, ε-PL, PVA/SPI and ε-PL/PVA/SPI composite nanofiber films.

**Fig. 4:**
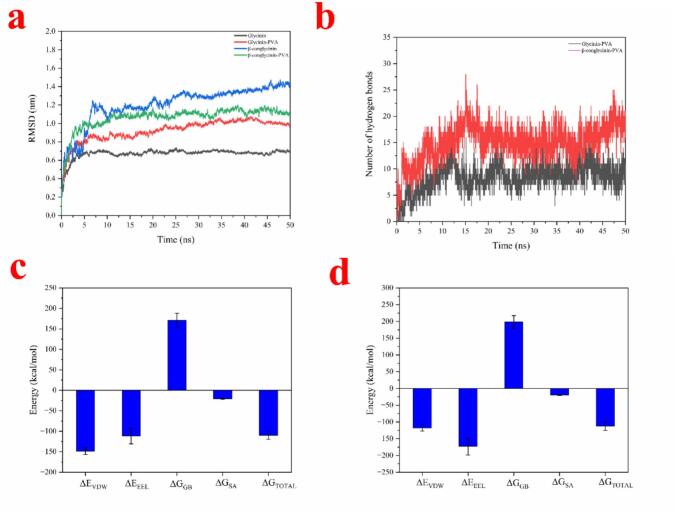
A. The RMSD of the four systems during MD simulation. B. The number of hydrogen bonds between PVA and glycinin and β-conglycinin during simulations. C and D. The binding free energies of PVA and glycinin (C) and β-conglycinin (D).

**Fig. 5:**
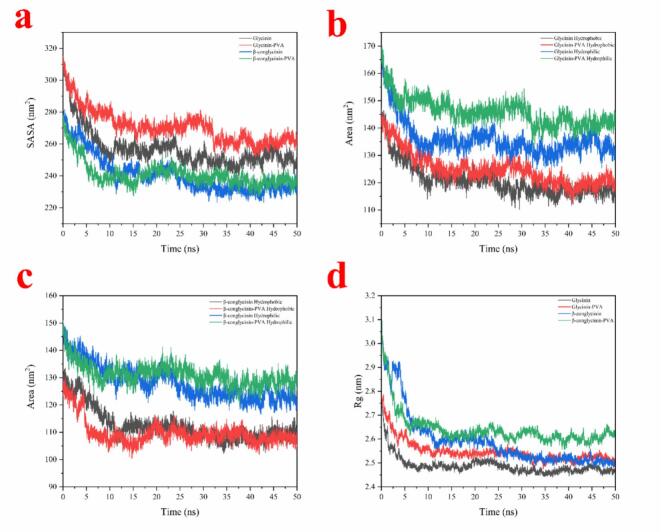
The change of SASA (a), hydrophobic and hydrophilic areas (b and c), and Rg (d) of glycinin and *β*-conglycinin during entire simulation. B—C. The change of of glycinin and *β*-conglycinin during entire simulation.

**Fig. 6:**
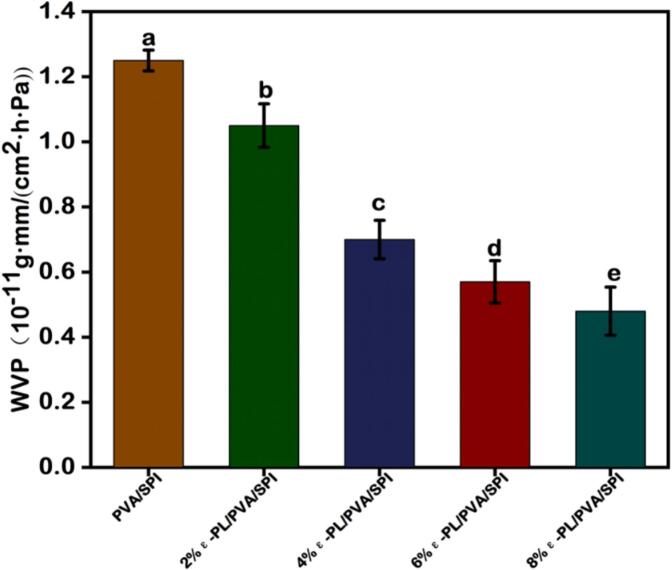
Effect of different concentrations of ε-PL on the WVP of nanofiber film.

**Fig. 7:**
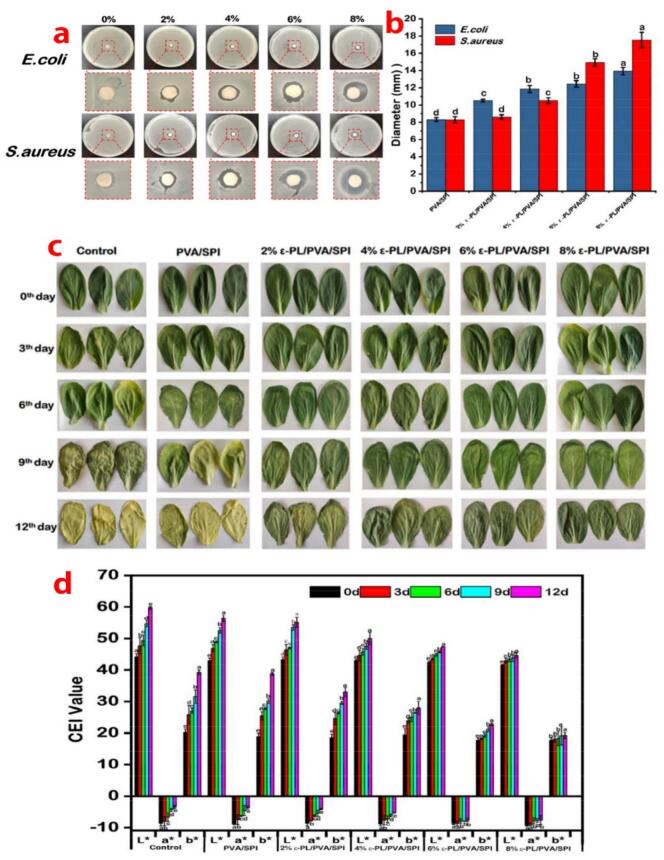
The inhibition cycle map of *S. aureus* and *E. coli* at different concentrations of ε-PL (a) and Diameter of antibacterial zone of PVA and PVA/SPI fiber films (b), the effect of different packaging materials on the physical appearance of Chinese cabbage leaves during storage period (c) and color changes (L^⁎^, a^⁎^, b^⁎^) in Chinese cabbage leaves during storage (d).

**Fig. 8:**
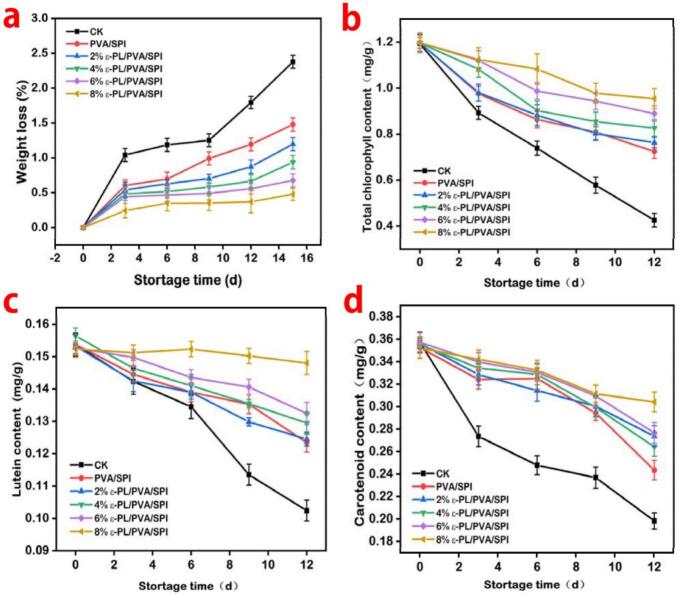
Changes in weight loss (a), total chlorophyll (b), lutein (c) and carotenoid (d) in Chinese cabbage leaves during the storage.

### 3.2 FTIR and XRD analysis of composite nanofiber films

The FTIR spectra of PVA, SPI, ε-PL, PVA/SPI, and 8 % ε-PL/PVA/SPI composite nanofiber films are shown in Fig. 3a. The PVA spectrum showed that the peak at 3440 cm^−1^ and 1629 cm^−1^ were attributed to the O—H bending vibrations and O—H stretching vibrations, respectively. In addition, the C—H stretching vibration peak was observed at 2925 cm^−1^, while the peak values of C—O stretching vibration were evident at 1058 cm^−1^ (Ma et al., 2024). The characteristic peaks of SPI were observed at 3403 cm^−1^, which was assigned to the N—H and O—H stretching vibration. Meanwhile, the stretching vibration of C—H was observed at 2927 cm^−1^. The peaks at 1658 cm^−1^ and 1542 cm^−1^ were attributed to the C=O antisymmetric stretching vibration of amide I and the N—H bending vibration of amide II, respectively. The C—N stretching vibrations and N—H bending vibrations in amide III were observed. The peaks at 1240 cm^−1^ represented typical superimposed absorption peaks (Li et al., 2020). Similarly, the ε-PL spectrum revealed that the characteristic peaks at 1670 cm^−1^, 1565 cm^−1^, and 1506 cm^−1^ corresponded to the C=O, N—H, and H-N-H stretching vibration. In the case of PVA/SPI nanofiber film, the intensity and position of characteristic peaks altered obviously when compared to PVA and SPI individually, indicating that an interaction existed between them. In the presence of ε-PL, PVA/SPI nanofiber film retained its characteristic peaks. However, the wavelength of the amide I and amide III was red-shifted, and the amide II mode was blue-shifted (Lan et al., 2020).

The structural formations of the PVA, SPI, ε-PL powders, and PVA/SPI nanofiber films containing different concentrations of ε-PL were analyzed using XRD (scanning range 5° ≤ θ ≤ 45°) to examine their crystalline components. The typical XRD patterns of various films are shown in Fig. 4. In this research, characteristic peaks for SPI appeared at 19.3°(2θ), ε-PL at 24.0° (2θ), and polyvinyl alcohol (PVA) at 19.3°(2θ) and 40.3°(2θ). Notably, the intensity of the characteristic peak of the ε-PL sample was lower than that of PVA and SPI, indicating that the ε-PL sample exhibited an amorphous character. The PVA/SPI film spectrum revealed a gradual decrease in crystallinity without the emergence of new peaks, and the characteristic peak of PVA at 40.3°(2θ) disappeared. The result showed that the compatibility between the PVA and SPI could modify biological macromolecule interactions, thereby influencing their crystallinity. In addition, no characteristic peaks were observed at 19.3°(2θ), but the intensity of the peaks at 19.3°(2θ) increased with higher ε-PL concentrations. The distinct appearance of these peaks indicated a degree of incompatibility among the macromolecules in the 2 % ε-PL/PVA/SPI, 4 % ε-PL/PVA/SPI, 6 % ε-PL/PVA/SPI, and 8 % ε-PL/PVA/SPI films. This incompatibility likely occurred owing to the improved crystalline regions of PVA/SPI/ε-PL film, which disrupted the packing of polymer chains, despite the increase in hydrophilic sites (Giteru et al., 2020).

### 3.3 Binding stability and mechanisms of PVA and SPI

To better understand the molecular mechanisms governing the binding of PVA and SPI, MD simulations were carried out to investigate the interactions between PVA and the primary components of SPI, namely soybean β-conglycinin (7S) and glycinin (11S). Firstly, the root mean square deviation (RMSD) analysis was conducted to estimate whether the simulation systems can reach equilibrium state. As depicted in Fig. 4a, on the whole, the fluctuations of RMSD values were less than 0.2 nm especially after about 15 ns, indicating that all four simulation systems achieved an equilibrium state. The representative binding conformations after 50 MD simulations were extracted as shown in Fig. S1 and S2. Overall, in agreement with experimental observations, the simulation results demonstrated that PVA can form a composite structure with SPI. According to a previous study, ε-PL was also demonstrated to form stable complexes with β-conglycinin (7S) and glycinin, which was primary driven by electrostatic and hydrogen bonding (Hu et al., 2023). Moreover, it can be observed that PVA could form hydrogen bonds via its hydroxyl groups with some amino acid residues of with β-conglycinin and glycinin. Collectively, it can be concluded that both PVA and ε-PL exhibit considerable binding stability with SPI, which is favorable for the construction of electrospun composite nanofiber films. Liu et al. reported that the addition of PVA improved the mechanical strength of the composite film because of the interactions between soy protein and PVA through hydrogen bonding and other interactions (Liu et al., 2017). As shown in Fig. 4b, PVA formed an average of sixteen hydrogen bonds with glycinin and nine with β-conglycinin after 15 ns of simulations. Similarly, hydrogen bonds were also found to play important roles in the interaction between PVA and other proteins, such as fish myofibrillar protein (Limpan et al., 2012) and zein (Xie et al., 2023).

Further, the binding free energy was determined to reveal the molecular mechanisms underlying the stable binding of PVA and SPI. As depicted in Fig. 4 c and d, the binding free energies (ΔG_TOTAL_) of the PVA-β-conglycinin (−112.51 ± 12.68 kcal/mol) and PVA-Glycinin (−110.09 ± 9.24 kcal/mol) are both negative, indicating spontaneous binding between PVA and SPI with strong binding affinity. The binding free energy of polymer-protein systems comprises four energy components, namely van der Waals energy, electrostatic energy, polar solvation energy, and non-polar solvation energy (Mahmoodi et al., 2022). Analysis of the four interaction types (Fig. 4c and d) revealed that the favorable contributions from van der Waals interactions (ΔE_VDW_), electrostatic interactions (ΔE_EEL_) and non-polar solvation energy (ΔG_SA_) outweighed the unfavorable effect of polar solvation (ΔG_GB_), synergistically stabilizing the PVA-SPI complexes. Among them, the van der Waals and interactions play an essential role in the binding process between PVA and both glycinin and β-conglycinin.

### 3.4 The solvent assessable surface area (SASA) and gyration (Rg) analysis

The hydrophobic and hydrophilic interactions were also reported to make a significant contribution to the binding of polymer chains and proteins. In comparison, the changes of SASA in the presence of PVA (Fig. 5a) implied the existence of hydrophobic and hydrophilic interactions during the formation of PVA-PSI complexes. According to Fig. 5b and c, it can be observed that the hydrophobic SASA decreased more slowly than the hydrophilic SASA in the presence of PVA. These results imply that the interactions between PVA and SPI mainly occurred in the hydrophilic part of the protein. Similar phenomenon was observed in the binding of ε-Polylysine and SPI, according to a previous study by Hu et al. (Hu et al., 2023). Rg is an important indicator of protein compactness and conformation change. The Rg of SPI during simulations were also determined as shown in Fig. 5 d. The results suggested that the binging of PVA slightly increased the Rg of β-conglycinin (from 2.471 nm to 2.495 nm) and glycinin (from 2.502 nm to 2.605 nm). It can be assumed that the changes of SASA were associated with conformational re-arrangements of SPI due to PVA binding.

### 3.5 WVP of composite nanofiber films

Generally, several factors affect the WVP of film materials, including film integrity, hydrophobicity, diffusion rate, solubility coefficient, the ratio of crystalline to amorphous regions, and density. The WVP of PVA/SPI nanofiber film was measured at 1.25 g mm/m^2^·h·Pa (Fig.6). The improved hydrophobicity and mechanical properties might be attributed to the interactions between the hydroxyl groups (-OH) of PVA and SPI, leading to the formation of hydrogen bonds and a decrease in the number of reactive -OH groups (Ma et al., 2024). After ε-PL was added to the system, the WVP of the resulting composite nanofiber film was significantly reduced (*P* < 0.05), and 8 % ε-PL/PVA/SPI exhibited the lowest of WVP, measuring 0.53 g mm/m^2^·h·kPa. Due to the strong interaction between the ε-PL and the PVA/SPI blends, the incorporation of anthocyanins contributed to a decrease in the water content of the films. In addition, the higher solubility and diffusion rates of the film increased their permeability, and the WVP results were consistent with those of solubility and swelling data.

### 3.6 Density, water content, and swelling of composite nanofiber films

Table 1 shows the density of PVA/SPI composite nanofiber films with different concentrations of ε-PL. The density of PVA/SPI nanofiber films was 0.19 ± 0.01 g/cm^3^. Compared with PVA/SPI nanofiber film, the density of composite nanofiber films increased after the ε-PL was added to the system (P < 0.05), with values ranging from 0.19 ± 0.01 g/cm^3^ to 0.35 ± 0.03 g/cm^3^. This increment is mainly attributed to the interspersion of ε-PL within the network structure of PVA/SPI, which increases the interaction between ε-PL and PVA/SPI polymer chains, resulting in closer molecular connections (Aydogdu et al., 2020). Generally, the swelling and solubility of the film are critical factors for determining water resistance. The swelling and solubility of a film mainly depend on the hydrophilicity and morphology of the substance (such as pores, crystal size, and crystallinity) (Almasi, Ghanbarzadeh and Entezami, 2010). As shown in Table 10, the solubility and swelling degree of PVA/SPI nanofiber film were 20.56 ± 0.47 % and 189.23 ± 13.14 %, respectively. After ε-PL was added to the PVA/SPI nanofiber film, both the swelling and solubility of the composite nanofiber film were significantly improved. PVA/SPI film containing 8 % of ε-PL exhibited the highest swelling and solubility, measuring 199.36 ± 23.54 % and 25.87 ± 0.3 %, respectively. Due to the addition of ε-PL to the PVA/SPI matrix, the content of the hydrophilic substance in the nanofiber film was increased, leading to larger aggregates in the PVA/SPI film and weakening the interaction between PVA and SPI (Liu et al., 2018). Consequently, the water absorption of the film increased, increasing the swelling and solubility of the film.

**Table 1:** Effects of different ε-PL concentrations on the density, water content, swelling, and solubility of the nanofiber films.

Films	Density (g/cm^3^)	Water content (%)	Solubility (%)	Swelling (%)
PVA/SPI	0.19 ± 0.01^a^	13.6 ± 0.22^a^	20.56 ± 0.47^a^	189.23 ± 13.14^a^
2 %ε-PL/PVA/SPI	0.20 ± 0.01^a^	13.3 ± 0.21^ab^	21.69 ± 0.29^b^	192.21 ± 21.32^b^
4 %ε-PL/PVA/SPI	0.21 ± 0.02^ab^	12.9 ± 0.16^c^	23.25 ± 0.36^c^	196.35 ± 23.10^c^
6 %ε-PL/PVA/SPI	0.23 ± 0.03^b^	12.5 ± 0.13^c^	24.36 ± 0.26^cd^	198.65 ± 22.23^cd^
8 %ε-PL/PVA/SPI	0.25 ± 0.03^c^	11.9 ± 0.15^d^	25.87 ± 0.31^d^	199.36 ± 23.54^d^

### 3.7 Preservation effect of Chinese cabbages

The PVA/SPI nanofiber film showed no obvious antibacterial activity against *S. aureus* and *E. coli*, as shown in Fig. 7 a and b. In contrast, the composite nanofiber film contained ε-PL exhibited excellent antibacterial activity, the diameter of the antibacterial zone gradually increased with the increasing concentration of ε-PL. Fig. 7b indicated that the higher the concentration of antibacterial agent, the stronger the antibacterial effect of nanofiber films, displays a dose-dependent manner between the ε-PL concentrations and the antibacterial zone diameters, with maximal antimicrobial efficacy achieved at 8 % (Liu et al., 2018).

Under the cold storage, the L^⁎^ and b^⁎^ values of Chinese cabbage leaf increased, while the absolute value of a^⁎^ decreased, indicating a lightening of green color and a gradually darkening of yellow as storage time extended, shown in Fig. 8c and Fig. 8d. The control group began to exhibit yellowing and wrinkling on the third day, and became inedible by the sixth day. This phenomenon is mainly attributed to chlorophyll degradation, which is due to the increasing chlorophyllase activity and elevated ethylene levels (Lee and Chandra, 2018). Compared with the control group and PVA/SPI packaged group, both the upward trend of L^⁎^ and b^⁎^ values and the downward trend of Chinese cabbage leaves became slower as the concentration of ε-PL increased. The experimental group showed only slight wrinkling by the sixth and ninth days. Moreover, Chinese cabbages packaged with 6 % ε-PL/PVA/SPI and 8 % ε-PL/PVA/SPI maintained their integrity effectively until the 12th day, and no significant signs of yellowing or wrinkling were observed. This observation indicated that the ε-PL embedded in the nanofiber film package maintain the appearance of Chinese cabbages, extending their shelf life.

### 3.8 Chlorophyll, carotenoids, and lutein changes of Chinese cabbages

The weight loss of Chinese cabbages under different treatments during storage was displayed in Fig. 8a. It can be clearly observed that the weight loss rate of control samples was higher than other groups on the 12th day, which might be because of the higher rate of evaporation and respiration (Aloui et al., 2014). In contrast, nanofiber films loaded with different concentrations of ε-PL remarkably reduced the respiration rate of Chinese cabbages leaves. The 8 % ε-PL/PVA/SPI composite film not only improved water resistance but also reduced the synthesis and activity of enzymes, thereby delaying the weight loss of Chinese cabbages during the storage period (Chitravathi et al., 2015).

Chlorophyll is the primary source of the green color in leafy vegetables and significantly influences consumer perception. During the aging process, chlorophyll degrades rapidly, leading to a reduction in its total content (Moncada et al., 2018). As shown in Fig. 8b, the total chlorophyll contents of all groups, including the control, SPI/PVA film packaging, and ε-PL/PVA/SPI films (2 %, 4 %, 6 %, and 8 % ε-PL), significantly decreased (*p* ≤ 0.05) throughout the storage period. The degradation of chlorophyll is mainly attributed to pH variations caused by the release of organic acids from vacuoles and chlorophyllase enzyme activity (Esturk et al., 2015). However, the chlorophyll content of the 8 % ε-PL/PVA/SPI group was significantly higher than those of the other groups (*P* < 0.05).

As shown in Fig. 8c, the lutein content decreased during the storage. On the 12th day of storage, the lutein contents in the samples packed with PVA/SPI and 8 % ε-PL/PVA/SPI were 0.10 ± 0.02 mg/g and 0.15 ± 0.04 mg/g, respectively. Generally, lutein is one of the predominant carotenoids found in green vegetables. The decrease in lutein content in the 8 % ε-PL/PVA/SPI was consistent with the result observed for carotenoid. Therefore, the quality of the leaf lettuce packed with 8 % ε-PL/PVA/SPI film during storage was superior to that of both the control and other packaging groups. These findings are consistent with those reported by Zehra et al. (2022)

As shown in Fig. 8d, the carotenoid content in Chinese cabbages decreased during storage. On the 12th day of storage, the carotenoid content in the control group was 0.19 ± 0.02 mg/g, while the contents in the PVA/SPI, 2 % ε-PL/PVA/SPI, 4 % ε-PL/PVA/SPI, and 6 % ε-PL/PVA/SPI packaged groups were 0.24 ± 0.03 mg/g, 0.26 ± 0.02 mg/g, 0.27 ± 0.04 mg/g, and 0.29 ± 0.03 mg/g, respectively. However, the carotenoid content in the 8 % ε-PL/PVA/SPI packaged group was notably higher at 0.31 ± 0.04 mg/g, indicating a slower depletion of the green color in collard greens by the 12th day of storage. This result suggested that the ε-PL/PVA/SPI composite film effectively delayed chlorophyll degradation and protected the carotene (pale yellow) from hydroxylated carotenoids (yellow) (Miceli et al., 2019).

## 4 Conclusion

The ε-PL/SPI/PVA composite film packaging material was successfully fabricated through electrospinning. The binding stability and interaction mechanisms of PVA and SPI were elucidated by MD simulations. PVA could form stable complex with SPI via its interactions with protein governed by hydrogen bonds and non-covalent interaction forces. Moreover, the effects of ε-PL on the properties of the nanofiber films were evaluated to optimize its suitable concentration. The results demonstrated that the 8 % ε-PL/SPI/PVA nanofiber film effectively maintained the physiochemical properties and organoleptic quality of Chinese cabbage during 12 d of cold storage at 4 ± 2 °C. The composite nanofiber exhibited the highest antimicrobial activity against *E. coli* and *S. aureus*. This study provides a new theoretical framework and insights into the fabrication of active biopolymer-based films for food packaging. Future studies should explore the potential of the ε-PL/SPI/PVA composite film in preserving other leafy green vegetables.

## CRediT authorship contribution statement

**Lanlan Wei:** Writing – review & editing, Writing – original draft. **Yanyan Yang:** Formal analysis. **Ziyi Qin:** Formal analysis. **Fuqiang Liang:** Data curation. **Hong Xie:** Funding acquisition.

## Declaration of competing interest

The authors declare that they have no known competing financial interests or personal relationships that could have appeared to influence the work reported in this paper.

## Data Availability

Data will be made available on request.

## Appendix A Supplementary data

Supplementary material Image 1
