# Supplementary material for: Fabrication of polyvinyl alcohol/soy protein isolate-based composite nanofilm for preserving Chinese cabbage
Source: Food Chem X. 2025 Sep 17;31:103048. doi: 10.1016/j.fochx.2025.103048 (PMC12495356; doi:10.1016/j.fochx.2025.103048)
Supplement: Supplementary file 1 — Supplementary material [file mmc1.docx]

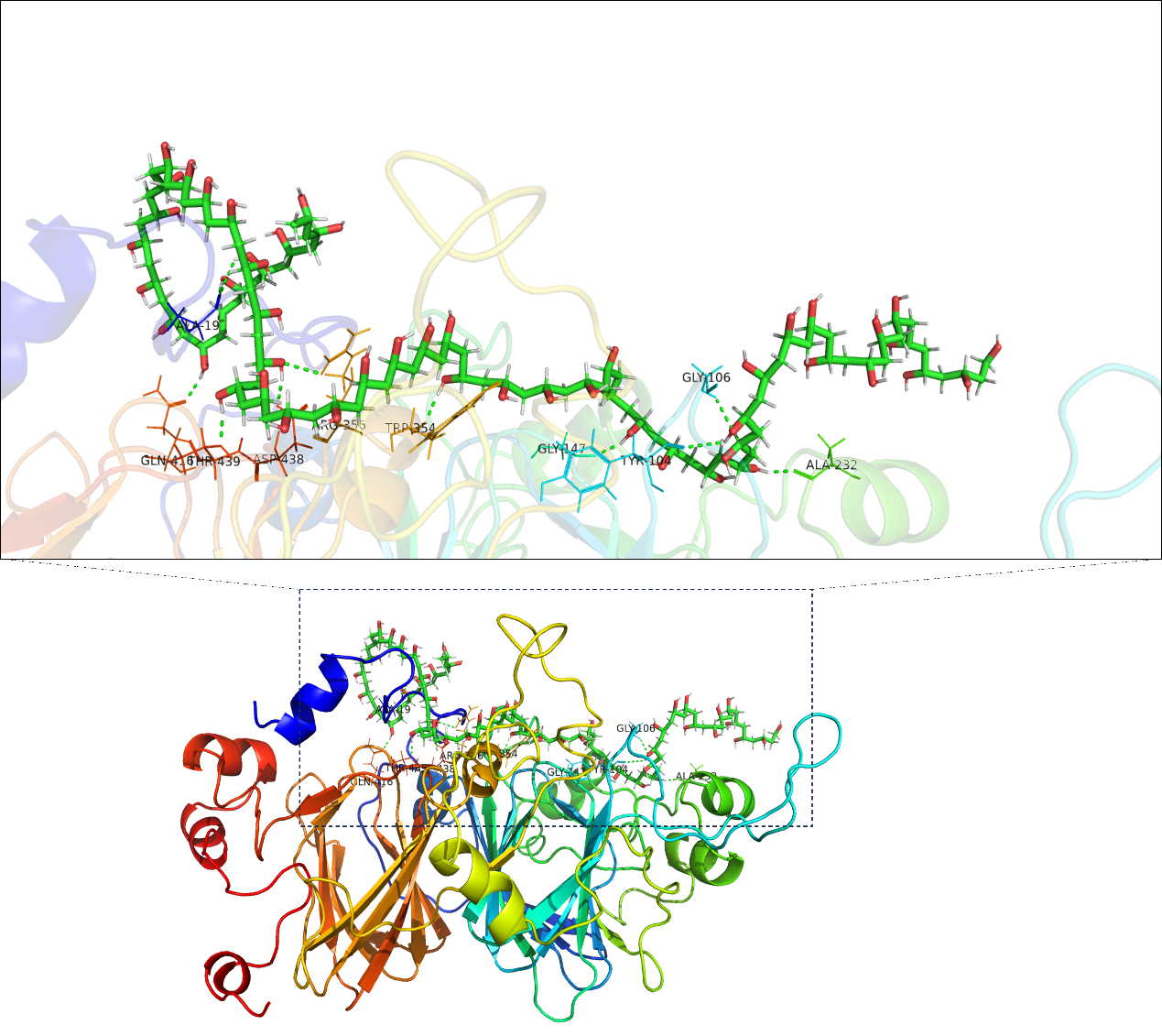


Fig. S1 The stable binding conformation and interaction mode between PVA and glycinin.


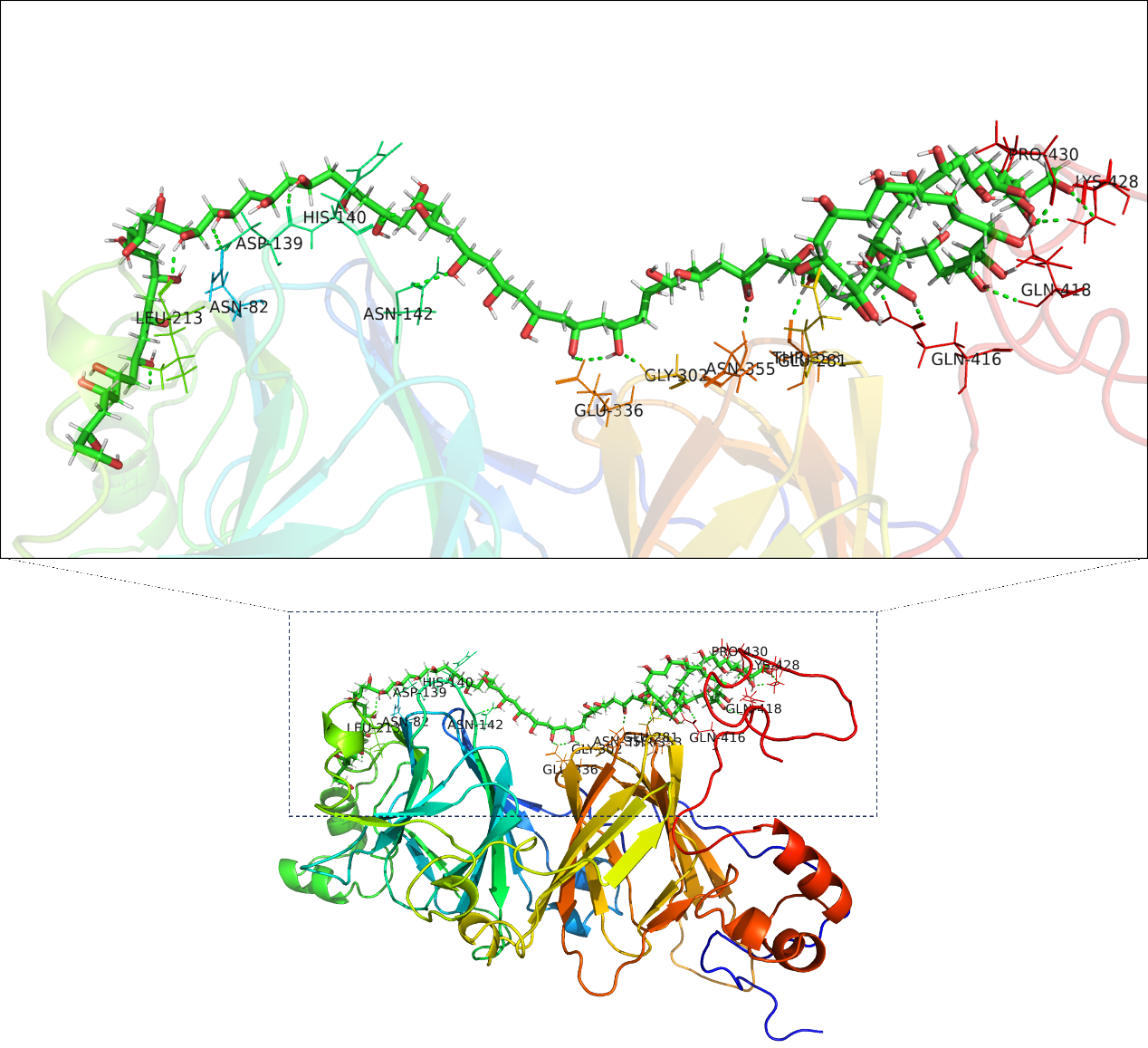


Fig. S2 The stable binding conformation and interaction mode between PVA and *β*-conglycinin.
